# APC licensing and CD4+T cell help in liver-stage malaria

**DOI:** 10.3389/fmicb.2014.00617

**Published:** 2014-11-11

**Authors:** Ian N. Crispe

**Affiliations:** Department of Pathology, University of WashingtonSeattle, WA, USA

**Keywords:** liver, hepatocyte, malaria parasite, immunity, CD4+ T cell help, CD8+ T cell, antigen-presenting cells, licensing

## Abstract

Malaria parasites spend a critical phase of their life cycle inside hepatocytes, in an environment with complex and distinctive immunological features. Here I will discuss how the immunological features of the liver and the adaptations of malaria parasites interact, resulting in defective CD8+ T cell immunity. These processes are explored with a focus on the mechanism by which CD4+ T cells deliver help to CD8+ T cells, and specifically through their interaction with antigen-presenting cells (APCs), resulting in “licensing” of the APCs and enhanced capacity to optimally activate CD8+ T cells. Synthesis of the available evidence supports a model in which the parasite-mediated manipulation of programmed cell death in infected hepatocytes impairs the capacity of the liver’s immune system to successfully license APCs and fully activate T cell immunity.

## INTRODUCTION

Malaria parasites are vulnerable to the mammalian immune system from the time they penetrate the skin as sporozoites, during the passage of these pre-hepatic forms through the blood to the liver, during their residence inside hepatocytes, and after their emergence, when they parasitize erythrocytes. In this review, I consider only immunity to the hepatocellular stage of the parasite. Liver-stage malaria parasites gain access to hepatocytes by crossing the liver sinusoidal endothelium, and both direct and indirect evidence suggests that they may exploit Kupffer cells, the liver’s large resident macrophage population, as portals to gain access to the underlying Space of Disse, and thus to hepatocytes (Frevert et al., 2005; Baer et al., 2007).

Once inside the hepatocyte, the malaria parasite establishes a vacuole, the membrane of which contains both host-derived proteins, and proteins secreted by the parasite. This parasitophorous vacuole separates the parasite from the host cell cytoplasm, and acts as the interface through which the parasite subverts cellular processes to protect it from host defense and to provide a supply of nutrients. The parasite’s interactions are highly adapted and successful. Thus, young children infected for the first time with malaria show little resistance, but over time and with repeated antigen exposure, immunity to the blood-stage gradually established sufficient control over parasite growth that the severity of clinical illness is reduced. However, it is not clear that repeated infection engenders any immunity against the liver-stage itself. Therefore, to study immunity to the liver-stage requires the use of animal models.

While natural immunity to the liver-stage is difficult to detect, artificial immunization protocols clearly generate such immunity, manifest both as reduced parasite load in the liver and as a delay, or the prevention of blood-stage infection. One approach is to infect a murine or a human subject with radiation-attenuated parasites (Hoffman et al., 2002). This approach results in sterilizing immunity in both species, and in mouse models a variety of host defenses have been implicated, including innate and antigen-specific immune cells (NK cells, CD4+ T cells, CD8+ T cells) and cytokines (IL-12, IFN-gamma; Doolan and Hoffman, 2000). The development of techniques to modify *Plasmodium spp.* genes has led to an alternative approach: the development of genetically attenuated parasites (GAPs). While human trials are only beginning (Spring et al., 2013), GAP can produce solid protection in mouse challenge models (Jobe et al., 2007; Trimnell et al., 2009; Vaughan et al., 2010). Such immunity correlates well with the capacity of T cells to secrete IFN-gamma, and with CD8+ T cells to kill parasitized hepatocytes *in vitro* (Trimnell et al., 2009; Epstein et al., 2011). In principle, a CD8+ T cell can manifest either or both of these effector functions *in vivo*; for example in viral hepatitis B, the major anti-viral effect appears to be due to IFN-gamma secretion, rather than cytotoxic killing (reviewed in Guidotti and Chisari, 2001). However, a recent *in vivo* study shows that GAP immunization, followed by boosting with a DNA vaccine, results in host defense that can destroy *in situ* hepatocytes that express malaria-encoded antigens (Chen et al., 2014). These data support the working hypothesis that the central effector mechanism against liver-stage malaria parasites is cytotoxic killing of the infected cells by CD8+ cytotoxic T lymphocytes (CTLs), rather than cytokine-driven intracellular cure of the infection.

## THE IMPORTANCE OF HELP

The fundamental immunological problem in liver-stage malaria is to account for the failure of effective immunity during natural malaria infection, but the capacity of irradiated sporozoites and GAP to induce such immunity. The role of CD4+ T cells in such immunity is complex. Direct antibody-mediated depletion of CD4+ T cells at the time of challenge does not impair anti-malaria immunity to the liver-stage, implying that at the effector level, CD8+ CTL are sufficient (Tarun et al., 2007). Yet experiments in animals that congenitally lack CD4+ T cells are consistent with the concept that CD4+ T cells are required to prime effective immunity (Overstreet et al., 2011). In terms of the biology of CTL maturation and memory, this makes complete sense. In host defense against intracellular bacteria, in anti-viral immunity, and particularly in CD8+ T cell responses against non-inflammatory antigens such as a minor histocompatibility antigens, CD4+ T cell help is essential, but the nature of the requirement for help depends on the nature of the antigen. Thus, minor histocompatibility antigens require CD4+ T cells to induce an effective CD8+ T cell response, while other antigens need CD4+ T cells to ensure either the long-term survival, or the memory effector function of CD8+ T cells (Prlic et al., 2007). There is a rough inverse correlation between the extent to which the antigen is accompanied by accessory signals, including pathogen-associated molecular patterns (PAMPs) and damage-associated molecular patterns (DAMPs), and the extent to which CD4+ T cell help is essential for a CD8+ T cell response. In viral hepatitis C, the main cause of immune failure and chronic infection appears to be the lack of a CD4+ T cell response, resulting in CD8+ T cells that become exhausted over time (Thimme et al., 2001; Grakoui et al., 2003; Radziewicz et al., 2007; Mueller et al., 2010). This same exhausted phenotype in CD8+ T cells is a major feature of immune failure in many potentially immunogenic cancers (Pardoll, 2012). These diverse diseases illustrate the central importance of CD4+ T cells in most CD8+ CTL responses; but their precise role in immunity to liver-stage malaria has yet to be defined.

As a complex parasite, *Plasmodium spp.* encode diverse non-mammalian proteins and synthesize many other non-mammalian molecules that could serve as PAMPs, but during the liver-stage these molecules could be sequestered inside the parasitophorous vacuole. However, this is clearly not the case for all CTL target antigens, since in the context of immunity induced by either irradiated sporozoites or GAP, infected hepatocytes can be killed. There is evidence that *Plasmodium spp.* may actively manage DAMPs, particularly those associated with cell death. Thus, infection of mouse hepatocytes with *Plasmodium yoelii* results in decreased p53, a mediator of G1 cell cycle checkpoint arrest, and a drug that sustained p53 expression reduced infection (Kaushansky et al., 2013a). The death of hepatocytes infected with wild-type (WT) parasites was driven by mitochondria and impaired by Bcl-2, while such control of host cell death appeared to be linked to the presence of a parasitophorous vacuole (Kaushansky et al., 2013b). Thus it seems that the parasite first delays the division of infected stressed hepatocytes, then inhibits their death, and finally allows them to die via the mitochondrial pathway of apoptosis. All of these maneuvers may sequester both DAMPS and PAMPs, eliminating pathways of immune activation.

Together with accessory signals from PAMPs and DAMPs, maturation of an effective CTL response with protective memory depends on CD4+ T cell help. In the case of malaria liver-stage antigens, the parasites reside in a cell type that expresses major histocompatibility complex (MHC) class I, but not MHC class II. Since CD4+ helper T cells are activated by antigen-presenting cells (APCs) that express MHC class II, it is clear that for CD4+ T helper cells to develop, malaria-encoded antigens must be presented by a different cell type, a mechanism termed cross-presentation; the T cell activation that follows is cross-priming (Bevan, 2006). Cross-presentation may occur by the uptake of cellular fragments or soluble proteins, or by the transfer of complete MHC-antigen complexes (termed: cross-dressing; Wakim and Bevan, 2011). In the liver, many cell types have the potential to take up antigen and engage in cross-presentation. Thus, there are liver-resident and circulating dendritic cells (DCs), Kupffer cells, liver sinusoidal endothelial cells (LSECs) and potentially also hepatic stellate cells (Ebrahimkhani et al., 2011). Among these, liver DCs and LSECs have the strongest credentials (Berg et al., 2006; Sumpter et al., 2007). Caveats concerning their role as APCs for liver-stage malaria antigens will be considered below.

## LICENSED VERSUS UNLICENSED HELP

Once a CD4+ T helper cell is activated, help may be delivered to a CD8+ T cell by several known mechanisms (Lee et al., 2003). Licensing of the APC occurs when the CD4+ T cell recognizes antigen presented by an MHC class II+ APC, and delivers activating signals through co-stimulatory pathways, involving such receptors as CD80, CD86, and CD40 on the APC and their counter-receptors on the T cell. These signals activate the APC, resulting it its capacity to deliver enhanced signals to any CD8+ T cell that recognizes antigen on the same APC (Smith et al., 2004). This mechanism, though elegant and precise, requires that two relatively rare cells, the antigen-specific CD4+ T cell and the antigen-specific CD8+ T cell, should find the same APC, but it does not require that they should do so at the same time. While recent advances in *in vivo* imaging clearly reveal sustained contact between both CD4+ T cells and CD8+ T cells with DCs, clear evidence for simultaneous clusters of all three cells is not a striking feature of active immune responses in lymph nodes (Germain et al., 2012). Licensing also clearly requires that the APC express MHC class I and MHC class II, while hepatocytes do not. Abundant evidence suggests that hepatocyte can act as APC and induce primary activation in naïve CD8+ T cells, both *in vitro* and *in vivo* (Bertolino et al., 1998; Klein and Crispe, 2006), but the lack of MHC class II expression means that a hepatocyte is ***an APC that cannot be licensed***, even in principle. Therefore if anti-malaria CD8+ T cells were to be primed directly on hepatocytes, they could only receive help through an alternative mechanism.

Two known possibilities exist to bypass the need for licensing. First, and most simply, the CD4+ T cell may secrete growth factors such as Interleukin-2 (IL-2) that promote CD8+ T cell proliferation and maturation. This model is supported by experiments in which CD8+ T cells, selectively deprived of the ability to respond to IL-2, fail to undergo full differentiation (Williams et al., 2006). The alternative mechanism through which CD4+ T cells can deliver help to CD8+ T cells is through a direct interaction that depends on CD40 on the CD8+ T cell, and appears to be a direct T–T interaction involving on CD40L on the CD4+ T cell (Isogawa et al., 2013). This mechanism of help allows for the CD8+ and the CD4+ T cell to interact with different APCs, and so could overcome the limitations of a CTL target antigen that is not readily cross-presented.

To understand how fully effective, long-lived CD8+ T cells might arise in response to *Plasmodium*-infected hepatocytes, it is important to know whether these CD8+ T cells are primed directly on hepatocytes, or via cross-presentation of antigen by an APC that can be licensed. There is some circumstantial evidence that cross-presentation of hepatocellular antigens is constrained. First, in experimental delivery of antigen to hepatocytes using an adeno-associated virus (AAV) vector, the activation of antigen-specific CD8+ T cells was exclusively via non-bone marrow-derived APC, consistent with direct-priming (Wuensch et al., 2010). Second, in a liver transplant patient infected with Hepatitis C, new CTL appeared that were restricted to the human leukocyte antigen (HLA) of the liver donor, implying that they were primed on a solid tissue and not on bone marrow-derived DCs (Lauer, 2005). Third, when hepatocytes undergo apoptotic death, a major pathway of their disposal is phagocytosis by other hepatocytes (Dini et al., 1992; Dalton et al., 2003), which would keep any pathogen-encoded antigens within the hepatocyte pool. These considerations render it plausible that hepatocellular antigens are limited in their susceptibility to cross-presentation.

However, these experiments are subject to the caveat that LSECs are non-bone marrow-derived APCs that are not replaced with host-derived cells after a liver transplant. They are efficient in antigen uptake, and fully competent to cross-present both hepatocyte antigens and cancer cell-derived antigens *in vitro* (Berg et al., 2006; Ebrahimkhani et al., 2011). Furthermore these cells express both MHC class I and MHC class II, and so could potentially be licensed. *In vitro*, the activation of CD8+ T cells by LSECs typically leads to tolerance, but this could be in their un-licensed state; antigen recognition by a CD4+ T cell on an LSEC could result in licensing, with a changed outcome and complete activation when a CD8+ T cell interacts with the same LSEC. Kupffer cells occupy a rather similar position, since they are MHC class I+ II+ macrophages, and a subset of them termed sessile Kupffer cells are both radiation-resistant, and persistent after a liver transplant (Kennedy and Abkowitz, 1997; Klein et al., 2007). *In vitro*, Kupffer cell also induce abortive T cell activation leading to tolerance, but explicit experiments to license these cells via CD4+ T cell help, and then test their capacity to prime CD8+ T cells, have not been attempted.

## THE ROOTS OF HELPLESSNESS

**FIGURE 1:**
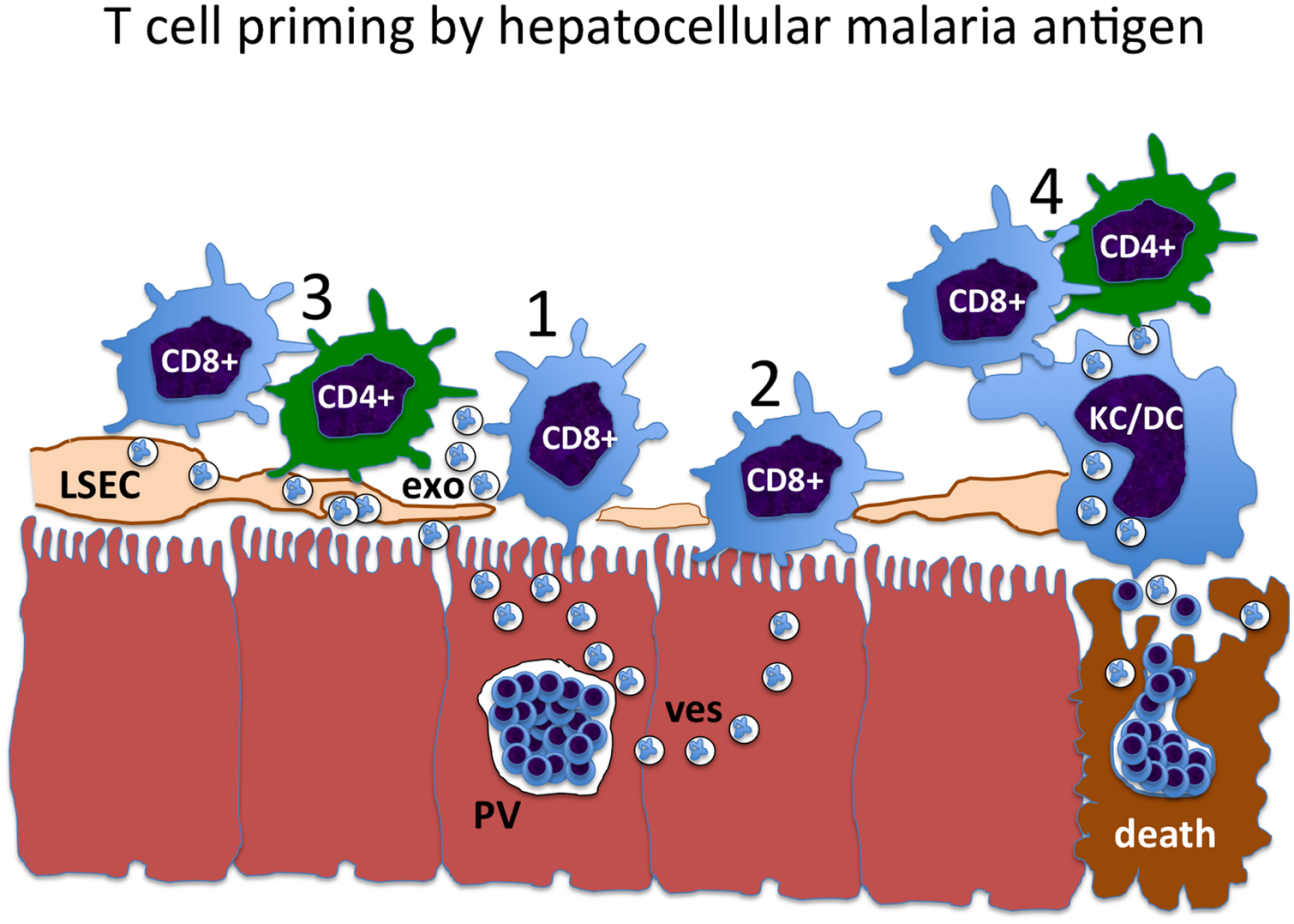
**The pathway of liver-stage malaria antigen presentation constrains the mechanism of CD4+ T cells help.** 1: Liver-stage antigens may exit from the parasitophorous vacuole (*PV*) in some kind of transport vesicle (*ves*), and be directly presented to CD8+ T cells by the infected hepatocyte. 2: Hepatocyte antigens may be taken up and cross-presented by neighboring hepatocytes, resulting in cross-presentation to CD8+ T cells. However, hepatocytes do not express MHC class II, and so cannot be licensed. This presents an obstacle to the delivery of CD4+ T cell help to these CD8+ T cells. 3: Malaria liver-stage antigens may be cross-presented by liver sinusoidal endothelial cells (*LSEC*), either by direct cell-to-cell transfer or via antigen-containing exosomes (*exo*). Since the LSEC expresses both MHC class I and class II, it can be licensed, facilitating CD4+ T cell help for the CD8+ T cell. 4: At the time of *death* of the infected hepatocyte and merozoite release, antigens may be cross-presented by myeloid cells, including liver-resident Kupffer cells (*KC*) and recirculating dendritic cells (*DC*). These cells also express MHC class I and class II, and are optimized to transmit CD4+ T cell help for CD8+ T cells through the licensing mechanism.

The mechanisms of direct versus cross-presentation, CD4+ T cell priming and the delivery of help via licensed APCs or otherwise collectively define the problem with respect to liver-stage malaria vaccine strategies (**Figure 1**). Any of the constraints that limit effective delivery of CD4+ T cell help to CD8+ T cells could apply in the context of natural malaria infection, but be circumvented by irradiated sporozoites or GAP vaccines.

First, viable malaria parasites could facilitate the death of the infected hepatocyte by a mechanism that favors the containment of PAMPs and DAMPs. Either irradiated sporozoites, or GAP that act as live-attenuated vaccines, may lose this ability. This would have the effect of rendering malaria antigen-specific CD8+ T cells highly help-dependent due to the limited PAMP and DAMP signals. Second, the hepatocytes that are infected by wild-type parasites could undergo death in a manner that facilitates uptake by neighboring hepatocytes. Hepatocytes are surprisingly phagocytic. They can take up activated CD8+ T cells and destroy them in a mechanism of tolerance termed suicidal emperipolesis (Benseler et al., 2011); and they can take up apoptotic hepatocytes through a mechanism that depends on the asialo-glycoprotein receptor (McVicker et al., 2002; Dalton et al., 2003). Such uptake would tend to contain parasite-encoded antigens within an APC that can engage CD8+ T cells, but cannot be licensed, resulting in impaired susceptibility to CD4+ T cell help. It is then possible that irradiated sporozoites or GAP result in hepatocyte death from a different mechanism, such as pyroptosis or necroptosis, that facilitates cross-presentation by other cell types. So far these issues have been considered in cancer, where many anti-cancer drugs kill cancer cells by diverse mechanism that may influence antigen presentation (Guo et al., 2014); but the principle is likely to apply also to infectious disease, in particular malaria parasites in hepatocytes.

In addition to regulation at the levels of antigen recognition and APC licensing, T cell immune responses, including the responses to many hepatocellular pathogens, are regulated through multiple classes of inhibitors molecules, including co-inhibitory signals such as PD-L1 and Galectin-9 that interact with counter-receptors (PD-1, and Tim3 respectively) on the T cells; and immune suppressive small molecules such as kynurenine, the product of tryptophan breakdown by the enzyme indoleamine 2,3-dioxygenase (IDO), and prostaglandin-E2, which is made in immunosuppressive macrophages by an enzyme cascade involving cyclo-oxygenase type -2 (COX2; reviewed in Crispe, 2014). Several of these mechanisms have already been implicated in resistance to blood-stage malaria infection (Butler et al., 2012), but their significance in regulating immunity to the liver-stage had not been documented.

Such co-inhibitory signals should not be seen as an alternative explanation to the limitations placed on CD4+ T help by constraints of cross-presentation and APC licensing. One striking example comes from virus hepatitis research, where the “exhausted” state of anti-hepatitis B virus (HBV) CD8+ T cells could be reversed by providing CD40, a key component of the licensing mechanism (Isogawa et al., 2013). Typically, CD8+ T cells in chronic HBV and HCV display all the features of exhaustion, and in both cases there is a lack of CD4+ T cell activation. An inclusive view is that the display of PD-1, Tim3, and Lag3 on the exhausted CD8+ T cell may be a downstream consequence of the inability to deliver CD4+ T cell help. In malaria, as in other globally important liver pathogens, it will be most important to understand whether this results from a failure of APC licensing, or the failure of those mechanisms that have evolved as back-ups in case licensing fails.

## CONCLUSION

Liver-stage malaria parasites inside hepatocytes occupy a cell type that can actively present antigen to MHC class I-restricted CD8+ T cells, but can neither directly activate CD4+ T cells, nor become licensed through interaction with CD4+ T cells. Since CD4+ T cell help is essential for full CD8+ T cell activation, function and protective memory, it is critical that both non-specific danger signals (DAMPs) and pathogen-associated innate immune activators (PAMPs) converge on an APC that can both cross-present antigen to CD4+ T cells, and be licensed to fully activate CD8+ T cells. In WT malaria infection, the parasite deftly subverts these mechanisms, in part through exerting control over the timing and mode of death of the infected cell. Successful experimental malaria vaccine strategies involve liver-attenuated parasites that furnish antigens, DAMPS and PAMPs, but fail to exert full control over the death of the infected cell. More detailed understanding of these mechanisms may lead to techniques to optimally deliver practical subunit vaccines, bringing CD4+ T cell help to anti-malaria CD8+ T cells and resulting in long-lived protective immunity at the liver-stage.

## Conflict of Interest Statement

The author declares that the research was conducted in the absence of any commercial or financial relationships that could be construed as a potential conflict of interest.
